# Stratigraphy of Fresco Paintings: A New Approach with Photoacoustic and SORS Imaging

**DOI:** 10.3390/jimaging9010016

**Published:** 2023-01-11

**Authors:** Francesca A. Pisu, Daniele Chiriu, Evgenia Klironomou, Giannis Zacharakis, George J. Tserevelakis

**Affiliations:** 1 Department of Physics University of Cagliari, Cittadella Universitaria, 09042 Monserrato, CA, Italy; 2 Foundation for Research and Technology Hellas, Institute of Electronic Structure and Laser, N. Plastira 100, 70013 Heraklion, Greece

**Keywords:** frescoes, photoacoustic imaging, Raman SORS, pigments stratigraphy, cultural heritage

## Abstract

Photoacoustic (PA) imaging is a novel, powerful diagnostic technique utilized in different research fields. In particular, during recent years it has found several applications in Cultural Heritage (CH) diagnostics. PA imaging can be realized in transmittance or epi-illumination (reflectance) modes, obtaining variable levels of contrast and spatial resolution. In this work, we confirmed the applicability of the PA technique as a powerful tool for the imaging of one of the most challenging artwork objects, namely fresco wall paints, to obtain precise stratigraphic profiles in different layered fresco samples. In this regard, we studied some multi-layered fragments of the vault of San Giuseppe Church in Cagliari (1870 AD) and some mock-ups realized specifically to test the potentiality of this technique. Due to complex structures of the frescoes, we used the Spatially Off-set Raman Spectroscopy (SORS) technique to provide complementary information. The experimental results were in agreement for both techniques, even for the three-layered complex structure, and were confirmed with Scanning Electron Microscopy (SEM) analysis of cross-sections. The combined use of these two techniques proved useful to investigate detailed hidden information on the fresco samples.

## 1. Introduction

Photoacoustic imaging offers several applications in different scientific branches, ranging from biomedical to Cultural Heritage (CH) fields [1,2,3,4,5,6,7], due to its capability to provide in-depth optical absorption contrast with high spatial resolution. In the field of conservation and artwork diagnostics, it has recently found several applications, such as revealing hidden underdrawings in paintings [8,9], discovering degradation and retouching features in historical oil paintings [6], and uncovering text in multilayered documents [10]. Recently it was also used for monitoring laser cleaning interventions on stonework [11,12].

Several techniques have recently been developed for the investigation of multi-layer CH objects, such as Terahertz Time-Domain Spectroscopy (THz-TDS), Optical Coherence Tomography (OCT), and Non-linear Optical Microscopies (NLOM). THz-TDS generally uses electromagnetic radiation with frequencies roughly between 0.1 and 10 THz, and, thus, provides no health risk or radiation damage to the object under study [13,14]. The THz technique shows very high penetration capabilities in opaque and turbid materials with satisfactory results, although generally expensive experimental equipment is necessary [15,16]. Another technique recently used for stratigraphic investigations, given its high transversal resolution, is Optical Coherence Tomography, an optical interferometric technique. It is a non-contact and fast technique, and does not require any preparation of the object under examination. Although its high axial resolution is around 1–10 um, it has a lower lateral resolution in the tens of micrometres, and its applicability is limited by the sample transparency to the used source [17,18].

Non-linear Optical Microscopies are new imaging techniques based on the use of femtosecond lasers, also recently applied in the field of cultural heritage [19,20]. One example of this new system is Multi-Photon Excitation Fluorescence (MPEF), involving simultaneous interaction with two or more photons from the laser excitation source with the sample, used for the characterization of thin painted surfaces, varnishes, and also, as proved in [21], for stratigraphic analyses of the thin painted surfaces of glasses. The NLOM in fact permits the obtaining of non-destructive 3D imaging of paintings with molecular and structural contrast.

Although all these techniques allow for high resolution imaging, photoacoustic imaging can be applied to a wide variety of samples and multi-contrast and multi-scale imaging can be achieved in a non-destructive manner. However, an immersion medium is necessary to increase the contrast of PA imaging.

Photoacoustic (PA) imaging is based on the absorption of a pulsed laser beam by a turbid material, with consequent thermal expansion and production of an initial local pressure rise (*p*_0_) proportional to the local laser optical fluence *F*, the dimensionless Grüneisen parameter *Γ*, the percentage of pulse energy converted into heat *η_th_* and the optical absorption coefficient for the employed wavelength *μ_λ_* [22], all of which is mathematically expressed by the following relation:

*p*_0_ = *Γμ_λ_*
*η_th_*
*F* (1)

The initial local pressure propagates in the form of ultrasonic waves prior its detection by an ultrasonic transducer (air-coupled or immersion), located behind the sample in transmission geometry, or on the same side with the laser beam in epi-luminescence (reflectance) geometry. The generated acoustic wave is typically found in the MHz frequency regime, with the detected amplitude directly proportional to the local absorption coefficient of the medium for the employed excitation wavelength [23]. In this manner, it provides excellent optical absorption contrast with high sensitivity.

**Figure 1:**
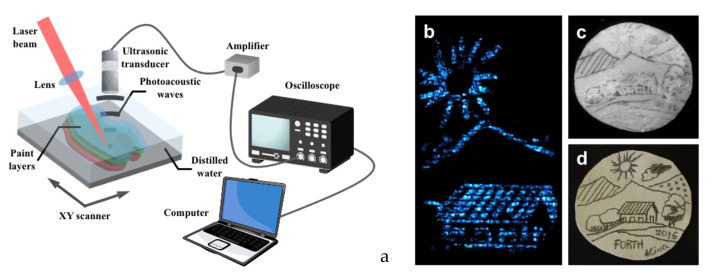
(**a**) Exemplificative PA setup; (**b**) detail of PA imaging obtained from a hidden drawing (figure adapted from [8]); (**c**) IR image in the hidden drawing; (**d**) original drawing.

The recorded acoustic wave can propagate in the materials and encounter low attenuation, up to about three orders of magnitude less than near-infrared optical radiation [3], permitting the receiving of information from deep layers located a few hundred μm, or even a few mm, below the sample surface. Figure 1 shows an exemplificative PA setup and some imaging results obtained from a hidden drawing.

Recent work [7] demonstrated the potential of the new epi-illuminescence geometry photoacoustic system for obtaining information on hidden graphite layers found in various wall painting mock-ups. In this paper, we further extended the capabilities of this new apparatus as regards the accurate reconstruction of the transverse profiles of real ancient fresco fragments, realized with the overlap of many pictorial layers, estimating the thickness values of the successive layers.

This study aimed to test, for the first time, the potential of the PA imaging technique in reconstructing stratigraphic profiles of thin and thick frescoed surfaces, moving from real fragments to mock-ups, also exploring the limits and the advantages of this diagnostic method in this challenging application. Furthermore, we attempted to combine PA imaging with the Spacially Offset Raman spectroscopy (SORS) technique [24,25,26], obtaining complementary information. SORS is another technique recently applied for stratigraphy in multi-layered samples [27,28], and is based on laser defocusing to receive information from the underlying layers. Despite the fact that the principles of SORS are entirely different from the PA techniques, they can potentially employ the same excitation source, offering the possibility to be integrated together into a single hybrid instrument for obtaining stratigraphic characterization of a sample. To reach this purpose, we compared the preliminary results obtained in our previous work [27] to those gathered in detail in this study. This test imposed the application of these techniques to fresco samples of which we knew all the structural characteristics. Beyond the easy realization of mock-ups, we opted for using samples with a real fresco structure, like the San Giuseppe Church vault fragments, preliminarily analyzed in [27]. In this direction, we experimentally demonstrated that the apparent information complementarity between PA and SORS diagnostic methods could be carried out in realistic cases of multi-layered fresco samples.

## 2. Materials and Methods

### 2.1. Samples

#### 2.1.1. Real Samples of Fresco

The three fresco fragments (called respectively F.01, F.02 and F.03) belong to a fresco of the San Giuseppe Church in Cagliari (ca. 1870 AD.) which has been unintentionally detached from the vault because of aging. All the samples appear to be constituted of different layers of plaster and painted on their top surface using different pigments. A 1 cm cross section along the depth direction was cut from one of the fragments to analyze the layer composition by surface Raman spectroscopy and scanning electron microscopy (SEM) imaging. Samples were analyzed at different points distributed on internal and external surfaces and they were not modified after recovery. The fragments were subjected to gentle dust cleaning with soft brushes. Table 1 summarizes all the samples with each point of analysis.

#### 2.1.2. Mock Up Samples of Fresco Paint

To test the PA performances in stratigraphic imaging, and provide a comparative study with the results obtained with SORS profiles, additional fresco mock-ups were realized. M.02 and M.07 had lime, sand, and calcium hydroxide substrates (the typical composition of a fresco, the “arriccio” and “intonachino” [29,30,31]) covered by lapis lazuli and cadmium orange (CdSeS), respectively. In addition, the other samples, V.02 and V.03, were made with compact earthenware. These were composed of different layers. The sample V.02 presented 3 layers, the bottom one made of graphite, the second of cadmium yellow (CdZnS), and then ochre. The sample V.03 was composed of a bottom layer made of ochre and two covering layers: one containing cadmium yellow and the other cadmium orange.

The different compositions of pigments used in the mock-up samples were selected to provide a complete scenario regarding the absorption properties of the PA source, in order to verify the contrast features of this technique in a multilayer structure. Actually, we used both compatible and non-compatible pigments with the fresco technique in order to get an overview of colors with low absorption properties in the Near Infra-Red (NIR), different from those already present in the real fragments of Table 1. The intention was to explore the limits of imaging PA using different pigments, of which an extensive chemical–physical and optical knowledge is already consolidated in literature.

### 2.2. PA Imaging Setup

The PA diagnostic system integrated a pulsed Nd:YAG laser (SL404, Spectron Laser Systems, Rugby, UK, with a maximum pulse energy 30 mJ, pulse width: 10 ns, pulse repetition rate: 10 Hz) emitting at an excitation wavelength equal to 1064 nm. The beam was attenuated and adjusted in a diameter of 1.2 mm, using an iris diaphragm to provide pulse energy less than 0.90 mJ on the plane of the sample. A 50 cm focal length converging lens was used to focus the radiation on a spot of ~1 mm, aiming at improvement of the imaging system’s sensitivity. The sample was placed on the bottom of a holder which was filled with distilled water, serving as a coupling medium for the propagation and efficient detection of the PA signal. The sample’s front surface was irradiated to generate laser-induced ultrasound from the highly absorbing layers. The resulting PA waves were transmitted through the painted layers and water prior to their detection in a reflectance configuration by a broadband, spherically-focused immersion transducer (HFM28, SONAXIS, Besancon, France; central frequency: 73 MHz; focal distance: 4.53 mm). The signals were subsequently enhanced by two radio frequency (RF) amplifiers (TB-414-8A+, Mini-Circuits, Camberley, UK; gain: 31 dB) providing a total gain of 62 dB, and, finally, digitized and recorded in the time-domain by a fast oscilloscope (DSO7034A, Agilent Technologies, Santa Clara, CA, USA; bandwidth: 350 MHz; sample rate: 2 GSa/s). The PA image was formed following raster scanning of the sample by a set of XY motorized stages (8MTF-75LS05, Standa, Vilinius, Lithuania), achieving a point-by-point data acquisition which was also synchronized with the laser pulse emission. The recorded waveforms were averaged two times to enhance the signal-to-noise ratio (SNR) values and transferred to a computer. A band pass filter. between 100 kHz and 30 MHz, was initially applied to eliminate high-frequency noise. Subsequently, the modulus of the Hilbert-transformed waveforms was estimated to reconstruct the imaged paint layers in three dimensions. Depending on the size of the samples, the scanning regions had dimensions ranging between 2 × 2 to 4.5 × 4.5 cm^2^, respectively, and were sampled, in all cases, using a pixel size of 300 × 300 μm^2^. The required total time for the acquisition of a PA image varied between 2.5 to 4 h. Both the control and synchronization of the developed PA apparatus were performed using custom-developed software, whereas the processing of the PA images was achieved through ImageJ and MATLAB.

### 2.3. Cross-Section SEM Images

Cross-section images were gathered by a scanning electron microscope, ESEM:FEI Quanta 200, under low vacuum conditions and in backscattered electron configuration. Cross-sections were obtained by embedding the samples in resin and lapping them until the desired grade of roughness was achieved. They were not coated with carbon or Au film, since the microscope was an Environmental SEM. The Low Vacuum regime ensured the analysis of these no-conductive samples. SEM images were obtained with 25–30 kV of high voltage at a working distance of 11 mm.

### 2.4. Raman Spectroscopy and SORS

Measurements were performed in ambient air at room temperature with a compact spectrometer B&WTEK (Newark-USA) i-Raman Ex integrated system with a spectral resolution of 8 cm^−1^. Near infrared micro-Raman scattering measurements were carried out in back scattering geometry with the 1064 nm line of an Nd:YAG laser. For each experimental setup, all the spectra were collected with an acquisition time of about 60 s (five replicas) and power excitation between 5 and 10 mW concentrated in a spot of 0.3 mm^2^ on the surface through a Raman Video Micro Sampling System (BAC151B), equipped with a 20× Olympus objective to select the area on the sample. Each measurement area represented a sampling surface of about 1 cm^2^. Micro-SORS measurements were carried out with the above Raman system by collecting the spectra in different sample positions. The SORS measurement was obtained by acquiring the spectrum of the surface and then operating a progressive defocusing to provide the contribution of sub-layers. Defocusing procedures were performed with the implementation of an automatized motion system realized with a micrometric motorized stage Opto Sigma HPS60-20X-M5, having motion resolution up to 1 μm. With the help of a sub-millimeter pinhole (diameter of 500 μm), the contribution of the upper surface (zero point) was isolated with respect to the sublayers. Then, after removing the aperture, spectral acquisition was performed at fixed defocusing distances (10 and 20 μm steps) from the zero position. Zero position spectrum and defocused spectra were finally subtracted to extract the Raman contribution at a specific defocusing distance. For each sample, the estimated thickness derived from an average of different cross-sections at different points of measurement and the errors were calculated as their deviation standards.

## 3. Results

### 3.1. Frescoes from San Giuseppe Church

**Figure 2:**
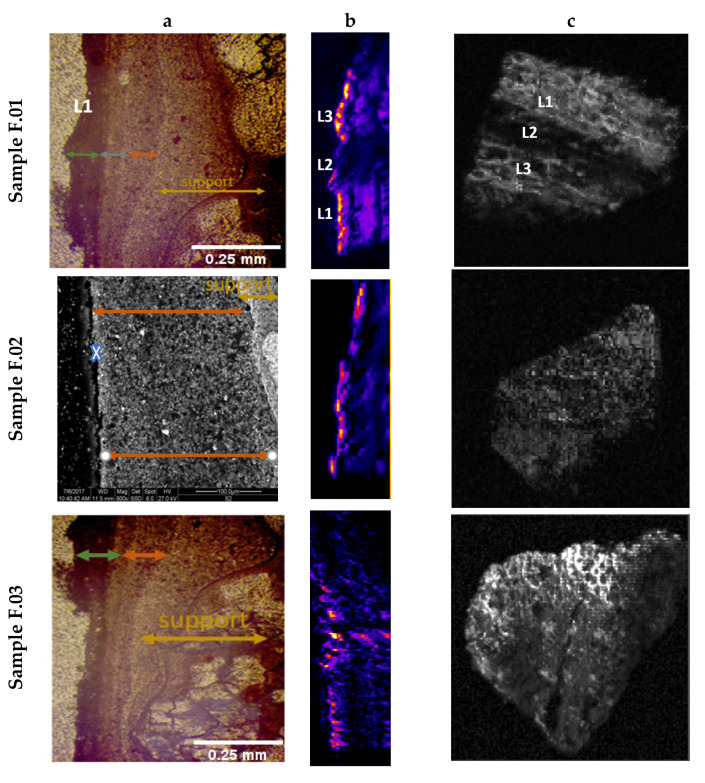
Moving from the left: (**a**) cross-section images; (**b**) cross-section imaging obtained with PA; (**c**) and relative PA later view images.

As the first step, SEM images were taken on different cross-sections of the samples (see Figure 2a) to confirm the presence of different layers. In the same figure we also proposed the cross-section imaging obtained with PA, Figure 2b, and the relative PA later view Figure 2c. A detailed analysis of these results is provided below.

Cross sections confirmed the presence of several pictorial layers, up to a maximum of three and a minimum of one, with variable thicknesses evaluated with different scales of magnification. The lime wall support (called intonaco) was not smoothed and homogeneous, making even the superficial brushstrokes inhomogeneous. The elemental compositions derived from Electron Dispersive Spectroscopy (EDS) are listed in Table 1 of [27] for L4 line (F.02 sample), in which higher atomic percentages were for Ca, Si, C, and O, a typical composition of a concrete and the amounts of Fe, Ca, S were due to the pigments of the painted layer. Their distribution in sample F.02 is shown in Figure 4a of [27].

**Figure 3:**
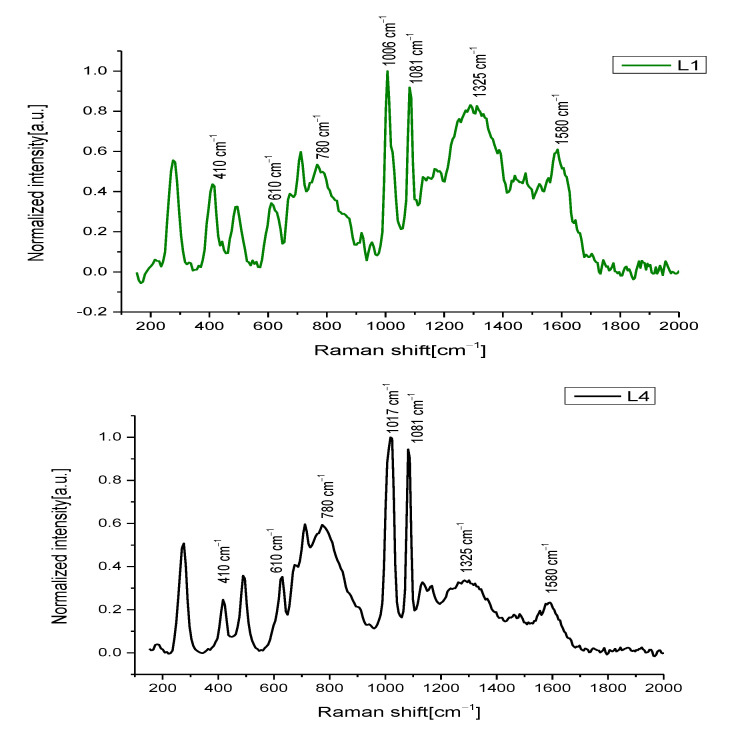
Raman spectra of different painted lines of fresco fragments.

Additional NIR Raman measurements were also carried out on fresco samples in order to individuate the chemical phases of the elemental analysis conducted by SEM–EDX. Raman spectra of L1 and L4 painted lines are shown in Figure 3. In detail, the 3 layers of the L1 line presented the following characteristics: the upper one was black, composed of amorphous carbon black (graphite), recognizable by the two characteristic Raman bands at 1325 cm^−1^ and 1580 cm^−1^ [32,33], mixed with calcite, having a strong peak at 1081 cm^−1^ (CO_3_ symmetric stretching [34,35]); the intermediate layer was a visible brownish/greyish color and was composed of a mixture of calcite, graphite, hematite (characteristic peaks at 286, 410, 614 cm^−1^ due to Fe–O symmetric stretching [36,37]), and gypsum with a strong band at 1006 cm^−1^ related to -SO_4_ symmetric stretching [38,39]; the last one was a mixture of hematite, calcite, calcium hydroxide (large band at 780 cm^−1^ [40]), and gypsum. Line L4 of the F.02 sample was composed of a single pink layer with the same composition of the just mentioned layer of L1. In this case, the pink hue was a mixture of hematite, calcite, calcium hydroxide, and gypsum (in anhydrous and hydrate phases).

The thickness results of the relative layers obtained from the SEM and optical images are shown in Table 2. The high error values associated with these thicknesses were due to the inhomogeneity of the fresco surface. The brush strokes were applied on a non-uniform intonaco substrate.

**Figure 4:**
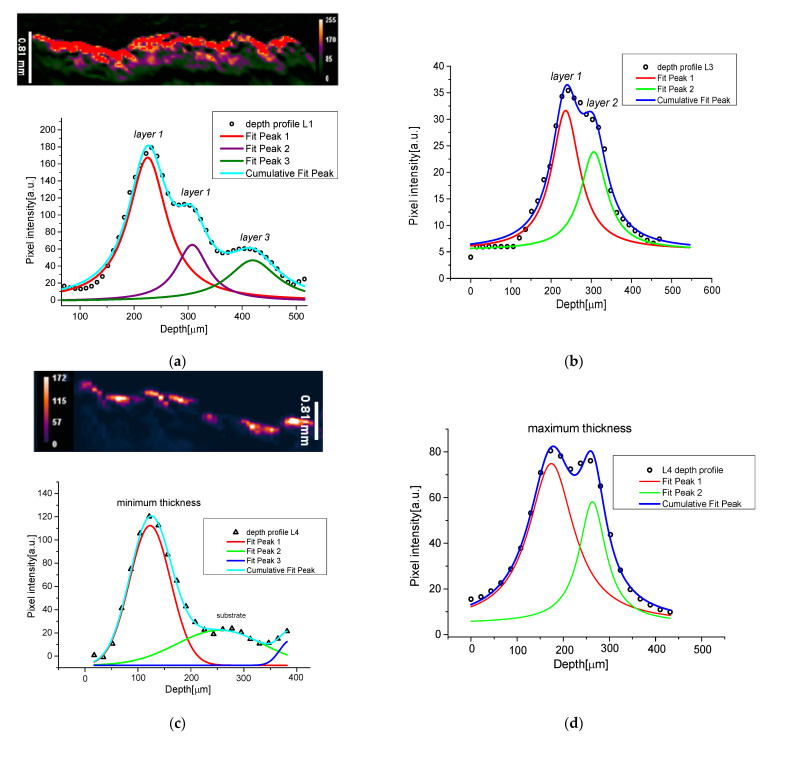
Stratigraphic profile fit obtained by PA imaging for sample F.01 the different painted lines: (**a**) z-profile of L1; (**b**) z-profile of L3; z-profile of L4 zone, maximum (**c**) and minimum (**d**) thickness evaluation.

After this preliminary characterization of the real fresco fragments, we concentrated our attention on the PA stratigraphic analyses. The experimental results were derived from an average of 20 depth profiles of the three different samples, obtained by ImageJ. To obtain the depth profiles we fitted the experimental data with Lorentzian and Gaussian functions, as displayed in Figure 4. From stratigraphic images of Figure 4, in comparison with Figure 2, we excluded the signals derived from a thicknesses below 1 mm of depth, from our analysis, because they were probably spurious signals due to the reflection of PA waves in the substratum. This assumption was confirmed by a detailed study of mock-ups realized with different bases (see next paragraph). As revealed by the deconvolution procedure in Figure 4a, the L1 painted line was composed of three layers of 60, 71, and 137 μm, respectively with a maximum error of 25%.

L3 was also made up of the overlap of two layers, as confirmed by cross-section images, with thicknesses assessed by PA techniques of 70 and 85 μm. The L4 thickness, evaluated in the second fresco fragment (F.02 sample) confirmed its composition of one layer. Due to the surface roughness of the sample, we estimated separately the maximum and the minimum thicknesses for this stratum, to decrease the associated absolute errors, obtaining 95 μm ≤ d ≤ 170 μm.

All the estimated PA values were consistent, and inside the error bands, with the SEM measurements shown in Table 2.

From the images of Figure 2c (lateral view), some particular areas, such as the L2 white line, presented a low PA signal that made it difficult to define an accurate z-profile in that region. For this reason, in our preliminary study, we focused our attention exclusively on the darkest painted surface areas providing high PA contrast. To have a complete characterization of sample F.01 and, thus, a stratigraphic analysis in the white area, we used the micro-SORS technique, recently applied for stratigraphy for CH [27,28] objects.

**Figure 5:**
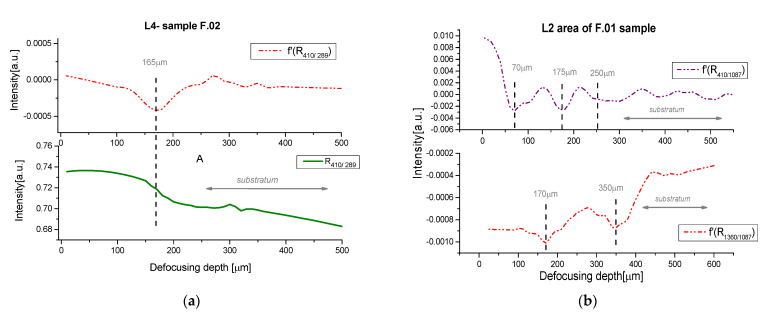
(**a**) Micro-SORS image of the sample F.02; (**b**) F.02 in the L2 zone (white).

For micro-SORS measurements, we employed the same excitation wavelength employed for PA imaging (1064 nm), to avoid fluorescence signals from the painted surface. For the micro-SORS technique, the investigation of fresco samples represented a challenging task. This was due to their inhomogeneous structures and the low-depth penetration in such turbid media, independently, on the selected laser wavelength and focusing objective. The results shown in Figure 5 were obtained by an average of five different z-profile points, as done for PA signals.

Firstly, we analyzed the F.02 sample made with one layer to make a comparison with the PA analysis. The depth profile was obtained following the decrease of the 410 cm^−1^ peak intensity, associated with the red pigment (hematite), with respect to the intensity of the band at 1087 cm^−1^ (calcite deriving from the substratum layer). To better visualize the variation associated with the interface of a new stratum, we estimated the first derivative of the z-profile. The thickness obtained for L4 was found at (165 ± 30) μm, in good agreement with the previous analysis.

Moving to the white layer of sample F.01, and looking for the SORS profiles, we followed different ratio intensities associated with each component of this layer to better identify the stratigraphy. Figure 5b offers the trends of hematite and graphite peaks at 410 cm^−1^ and 1360 cm^−1^, respectively, with respect to the principal band of calcite at 1087 cm^−1^. For the sake of brevity, we reported only the first derivative of the analysis. From the hematite peaks, the obtained profile showed three layers at 70, 175, and 250 μm, with thicknesses of about 70 μm, 100 μm, and 75 μm, respectively. Regarding the graphite profile, two layers were visible at 170 and 350 μm, but the simultaneous presence of this compound in two adjacent layers meant the identification of their profiles was more difficult. This specific problem is not so unusual in painted artworks. For this reason, it was necessary to reconstruct the stratigraphic information by combining the information from diverse profiles.

### 3.2. PA and SORS on Fresco Mock-Ups: Comparing Limits and Applicability

With the intent of validating the PA imaging methodology and exploring its limits or advantages on fresco samples, different fresco mock-ups were realized. In Figure 2b, where the PA transversal view imaging is shown, three separate signals with the same trend, but a decrease in intensity, are visible. This condition could be associated to spurious wave reflection phenomena in the fresco substratum with respect to the real signal of the painted surface (higher contrast in the image). In fact, in the fresco fragments, the substratum was composed of sand grains with variable sizes, which could produce spurious oscillation of the PA signal derived from the surface.

To verify this assumption, and exclude the two signals below the brighter one in our analyses of depth, we reproduced two different typologies of fresco mock-ups: the first one made with the original substratum (sand, calcium hydroxide, calcite), similar to the real fresco fragments of Table 1, and the second one made with a homogeneous substratum of compact earthenware. Both the mentioned typologies were realized with different painting structures, as reported before in Table 1 for samples M02, M07, V02 and V03.

**Figure 6:**
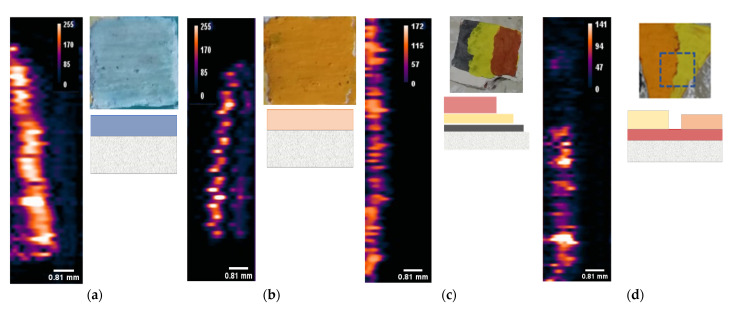
PA imaging of transverse view of (**a**) M02; (**b**) M07; (**c**) V02; (**d**) V03 samples with relative pictures and respective cross-section sketches.

The different PA profile images obtained from these mock-ups are shown in Figure 6. In particular, in Figure 6a,b the samples made with an original fresco substrate (the “arriccio”) presented another shade line below the sharp one, in contrast with those obtained in the V.02 and V.03 samples (see Figure 6c,d). This finding confirmed the hypothesis that the second signal was due to some artifacts of the PA waves originated by the presence of size variable substratum grains. The intensity of this spurious signal seemed to be proportional to the absorbance and the thickness of the top layer. In order to avoid the contribution of these spurious signals, and to present an analysis on multi-layered samples, we focused our attention only on the second group of samples, V.03 and V.02 (made from a succession of up to 3 layers). For these samples we also executed a comparative analysis with SORS profiles.

Actually, in this way, we wanted to test the PA stratigraphic capability for thick-painted layers, obtained with pigments presenting higher or lower absorbance in the NIR region. These pigments were spread with larger thicknesses, unlike the real sample analyzed above (<200 μm), to demonstrate the maximum depth that could be achieved using the PA signal. All PA measurements were compared with SORS results obtained in the same analyzed regions to provide complementary information about the stratigraphy.

**Figure 7:**
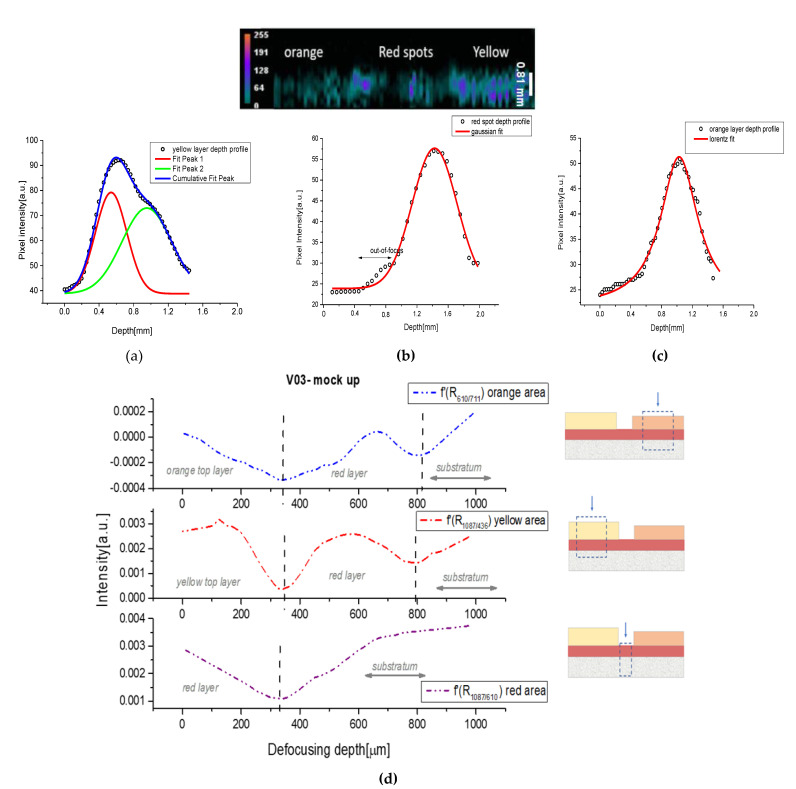
Layers fit of V.03 sample obtained from PA stratigraphic images for: (**a**) the yellow zone; (**b**) the red spots from the bottom layer; (**c**) the orange zone; and (**d**) SORS stratigraphy obtained for the same areas.

Sample V.03 was made with two different cover layers, obtained with Cd-yellow and Cd-orange pigments. Below them, a red layer of ochre was present, and in the middle of the sample, some areas of this red layer were left visible. The obtained PA transversal image was noisy, and the contrast was not very strong (Figure 6d and Figure 7), since the two colors chosen for the cover layer did not absorb enough in the NIR region, but it was still possible to see the difference in intensity between the orange and yellow areas. In fact, the latter showed greater intensity, due to its higher NIR absorbance. The two cover layers had respective depths of 500 μm for the yellow and 400 μm for the orange. The red spot belonging to the bottom layer showed a PA profile with a maximum peak shifted to about 500 μm, compared to the signal of the top layers, in agreement with its real position.

However, since the very low signal associated with the orange layer hindered the estimation of the PA profile, we obtained only a curve with a medium value of 550 μm (Figure 7c). Higher signals were generated from the yellow stratum, through which we were able to receive information, and also from the bottom layer, as shown by the deconvolution procedure in Figure 7a. In this case, we obtained a thickness of 490 μm for the yellow layer, and an estimation of 600 μm for the substratum composed from the union of the red layer and the intonaco.

A comparison with the SORS profiles was immediately possible by studying the graph of Figure 7d, in which, for the sake of brevity and clarity, we put only the first derivatives of the depth curves. In this case, we followed different relative ratios based on the compound present on the surface. For example, in the yellow area, we normalized the spectra with a characteristic peak of CdS and we followed the distribution of calcite (1087 cm^−1^), obtaining an average thickness of 400 μm for both the layers, yellow and red. In the red spots, we found only a minimum associated with the red layer with an average thickness of 300 μm. The orange zone revealed two minima, respectively, around 370 and 800 μm. Regarding the yellow top layer, the obtained value underestimated the real thickness, but it should be noted that the gathered information was derived from the average of different sampling points (inhomogeneous surface) and the combination of different compound profiles (different ratios). In addition, the SORS profile was linked to the depth of “defocusing”, which did not always match the exact thickness of the sample under examination, as is well explained in the literature [24,25,26,27,28].

**Figure 8:**
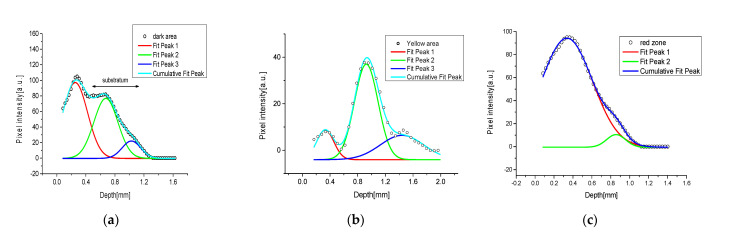

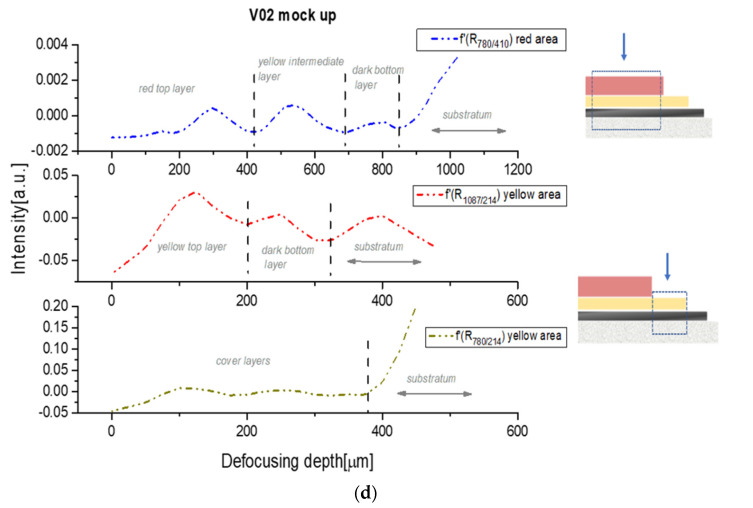
Layers fit of V02 sample obtained from PA stratigraphic images for: (**a**) the dark area, (**b**) the yellow area, (**c**) the red area; and (**d**) the same profiles obtained with the SORS technique.

Regarding sample V.02, PA and SORS profiles showed, on the whole, a bottom dark zone composed of only a layer of graphite (around 250–350 μm), in the middle a yellow area composed of two layers, the graphite seen before, and a cover layer of yellow cadmium of around 200–300 μm. Finally, the red area was composed of the previous yellow layer covered by a stratum of red ocher (about 500–600 μm). Their deconvolutions are shown in Figure 8.

In particular, as seen before for sample V03, the yellow cadmium was not a good absorber of the NIR excitation source (very low pixel intensity associated with its signal), even if, as seen in Figure 8b, an identification of its thickness and the thickness of the dark layer below it was possible. In this case we obtained 300 μm for the yellow stratum, 350 μm for the dark one, and a broad band of around 740 μm for the white substratum.

In Figure 8a the dark zone was analyzed obtaining around 330 μm for the painted layer and a broad signal composed of different bands associated with the substratum until a maximum of 800 μm. For the red area (see Figure 8c), the deconvolution showed two peaks, an intense one of about 600 μm, associated with the red layer, and a very weak shoulder for the underlying yellow layer of around 230 μm. No further information on other underlying layers could be obtainable from these PA images.

In Figure 8d we proposed the SORS stratigraphic results obtained from the same area discussed before. Following the intensity ratio between the calcium hydroxide (780 cm^−1^) and the hematite (410 cm^−1^), and starting the defocusing from the top red layer, we obtained three pigmented layers with average thickness values of around 500 μm (red), 200 μm (yellow) and 150μm (dark), followed by a white substratum, which started below 830 μm of depth. The values obtained with the SORS technique were more accurate than the PA ones in this sample, even if there was a slight underestimation of the real thickness of the red layer, which could be explainable by the inhomogeneous surface.

These preliminary results showed that, even for thicknesses of more than 200 μm, we could estimate the fresco stratigraphy with PA. Their accuracy decreased as a function of the layer thickness (over 400 μm) and the absorbance at the source wavelength. The final result became quite approximate, especially when the surface signal was weak. However, the combined use of SORS, even in such thicknesses, made it possible to obtain information with a fair degree of accuracy, overcoming the PA limits encountered in some mock-up compositions.

## 4. Conclusions

In this work, we sought, for the first time, to explore the photoacoustic (PA) imaging potential, using an excitation wavelength at 1064 nm, to reveal the stratigraphy of highly turbid and inhomogeneous materials, such as frescoes, and also combining it with micro-SORS, using the same wavelength. The results of the PA on real samples were also complemented by studies on mock-ups of different thicknesses and realized with different materials, to increase the information recovery of this novel approach and to validate the obtained results. Although the frescoes were not homogenous and were characterized by grains of different sizes, which could cause reflections of the main signal, an estimation of the stratigraphy of the painted surface was possible, and when the PA could not work properly, due to low absorbance from the surface pigments, the SORS technique could easily be used. This work is presented as a preliminary study on some fresco mock-ups with the future aim of increasing the variability of the fresco samples and, systematically, their thicknesses to achieve a maximum limit of the PA imaging accuracy. In addition, to overcome the limits encountered in this study, different excitation wavelengths could be employed to investigate layers presenting variable optical absorption and structural properties. Finally, a future goal is to extend the versatility of the technique to other painting surfaces, such as canvas and pictorial panels, and combine it with other imaging techniques, such as multi-photon excitation fluorescence, to obtain full knowledge of a sample under examination.

## Figures and Tables

**Table 1:** Real fresco samples and mock-ups.

Fresco Fragments from San Giuseppe Church			
Sample	Substratum	Painted Region or Line	N° of Layers
F.01 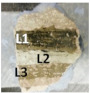	Mortar, calcium hydroxide	L1	3 layers
		L2	2 layers
		L3	2 layers
F.02 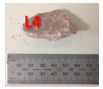	Mortar, calcium hydroxide	L4	1 layer
F.03 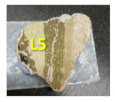	Mortar, calcium hydroxide	L5	3 layers (same composition of L1)
**Fresco Mock-ups**			
**Sample**	**Substratum**	**Painted Area or Line**	**N° of Layers**
M.02 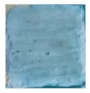	Lime, sand, calcium hydroxide	Total area	1 layer: lapis lazuli
M.07 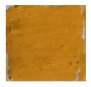	Lime, sand, calcium hydroxide	Total area	1 layer: cadmium orange (CdSeS)
V.02 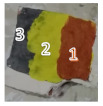	Compact earthenware	Area 1	3 layers: graphite, cadmium yellow (CdS) and ochre
		Area 2	2 layers: graphite, cadmium yellow
		Area 3	1 layer: graphite
V.03 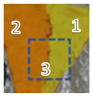	Compact earthenware	Area 1	2 layers: ochre and cadmium yellow
		Area 2	2 layers: ochre and cadmium orange
		Area 3	1 layer: ochre (between yellow and orange)

F.01 was composed of L1, L2, and L3 painted lines having different stratigraphy: L1 was dark at the top, L2 was white and L3 brownish-grey. L1 was composed, on the whole, of three layers, including the dark one, while L2 and L3 presented two layers, the visible one on the surface. Sample F.02 was composed of only one layer called L4. In sample F.03 the only line analyzed was the brownish line, labeled as L5, equal in composition and structure to the L3 line of F.01.

**Table 2:** Thickness measurements of different layers of fresco samples by SEM and PA imaging.

**SEM analysis**					
* **L1** *			* **L3** *		* **L4** *
**1st layer** [μm]	**2nd layer** [μm]	**3rd layer** [μm]	**1st layer** [μm]	**2nd layer** [μm]	**1st layer** [μm]
$\left(81\pm 30\right)$	$\left(60\pm 20\right)$	$\left(80\pm 30\right)$	$\left(81\pm 30\right)$	$\left(60\pm 20\right)$	$\left(107\pm 50\right)$
**PA analysis**					
* **L1** *			* **L3** *		* **L4** *
**1st layer** [μm]	**2nd layer** [μm]	**3rd layer** [μm]	**1st layer** [μm]	**2nd layer** [μm]	**1st layer** [μm]
$\left(60\pm 15\right)$	$\left(71\pm 20\right)$	$\left(137\pm 30\right)$	$\left(70\pm 21\right)$	$\left(85\pm 20\right)$	$d_{max}=\left(170\pm 40\right)$ $d_{min}=\left(95\pm 15\right)$

## Data Availability

Not applicable.
